# The Cholera

**Published:** 1892-10

**Authors:** 


### HALL'S JOURNAL OF HEALTH

TRUTH DEMANDS NO SACRIFICE; ERROR CAN MAKE NONE.

Vol. 39. OCTOBER, 1892. No. 10.

### THE CHOLERA.

The consternation consequent upon the threatened invasion of our territory by this most dreaded scourge, has almost died away, the prompt and efficient action of our City authorities having met the dangerous intruder at the outer gates, and to all present appearance, vanquished him.

Its fatal ravages in Russia were sufficiently alarming to put the nations of Western Europe on their guard, and, without exception, extraordinary precautions were resorted to prevent the spread of the disease beyond the Russian frontier. That it reached St. Petersburg is an admitted fact, and there was a wide spread alarm there, causing very many of the well to do classes to hastily pack up and depart the City, active business in many quarters being almost wholly suspended.

The writer obtained a forced acquaintance with this unwelcome visitor during its invasion of Cincinnati in the year 1848. Many of his friends were stricken by the plague and died, some in his presence and under his watchful care. Night after night he gave them what aid and comfort he could, and thus became familiar with the attack and progress of the disease, even to its too frequent fatal termination.

It was a fierce struggle with its victims whose life currents ran out with unquenchable rapidity. The entire city was as a house of mourning, wherein master and servant were alike overpowered and helpless. The bereaved of that day can well understand and appreciate the necessity for the adoption of a rule of defense as severe as the exercise of martial law in the military crisis, and yet there is some measure of excuse for the resistance of our Long Island neighbors to the establishing of an extensive Cholera Hospital in such nearness to their year-round habitations as Fire Island. Panic stricken, they resorted to forceful means to prevent it, but happily the great dread of deadly contagion appears to have abated, and peace and quiet have regained their wonted sway.

Persons overcome by fear, are most likely to imbibe the poison germs with which the air in infected regions is charged, for it produces a state of receptivity in the system which invites disaster.

A high medical authority makes the following statement, "A number of friends, several of them doctors, and familiar with India, the home of cholera, were discussing that disease ; and though their views differed on many points, they were unanimous in believing, first, that the chief vehicle of cholera-poison is water ; next that the time of chief peril and susceptibility is before breaking fast in the morning, insomuch that an early draught of water is almost deadly, and even the act of brushing the teeth is the most dangerous deed of the day. They further agree that the danger was almost entirely removed by the simple precaution of eating something, or even drinking something with a decided flavor, such as coffee, before allowing a drop of plain water to pass the lips. No one contended for any thing but the simple facts, though it was, of course, not difficult to account for them by a neat scientific theory. Thus, it is widely accepted in some quarters that the comma bacillus is the true microbe of cholera ; it is, moreover, granted that this fungus is so susceptible to even a weak acid that it immediately perishes when in contact with the gastric fluids. Now, the acid fluids of digestion are not always present in the stomach, but are at once provoked by the presence of any kind of food material whatever. The only exception to this law is found in the case of tasteless (but not necessarily pure) water, which passes straight through the stomach without provoking a flow of digestive acids. Hence the danger of drinking plain water on an empty stomach ; hence, too, the security of taking some tea and a biscuit, or any similar trifle, the first thing in the morning. If once the virus can shoot safely through the stomach and get into the small intestine, it is henceforth quite at home and can multiply indefinitely and poison the system with its deadly ptomaine. If so simple an expedient as eating a bun will erect an effective barrier to the passage of the microbe, by all means let the bun be eaten. Fruit may be cooked, milk boiled, and water filtered, and ice eschewed, and cleanliness fostered, and any other measure adopted which will quiet nervousness and allay fear. Fear has a well-known influence on the alimentary canal which is very remarkable, and reduces it to a condition in which it can neither do its work or repel invasion."

It will not be wise to relax in cautionary measures. The danger is not all passed.

